# A Review: The Potential Involvement of Growth Arrest-Specific 6 and Its Receptors in the Pathogenesis of Lung Damage and in Coronavirus Disease 2019

**DOI:** 10.3390/microorganisms11082038

**Published:** 2023-08-08

**Authors:** Daria Apostolo, Luciana L. Ferreira, Alice Di Tizio, Barbara Ruaro, Filippo Patrucco, Mattia Bellan

**Affiliations:** 1 Department of Translational Medicine, University of Piemonte Orientale (UPO), 28100 Novara, Italy; daria.apostolo@uniupo.it (D.A.); luciana.ferreira@uniupo.it (L.L.F.); 20036893@studenti.uniupo.it (A.D.T.); mattia.bellan@med.uniupo.it (M.B.); 2 Respiratory Diseases Unit, Medical Department, AOU Maggiore della Carità Hospital, 28100 Novara, Italy; 3 Pulmonology Department, University of Trieste, 34128 Trieste, Italy; barbara.ruaro@yahoo.it; 4 Division of Internal Medicine, Medical Department, AOU Maggiore della Carità Hospital, 28100 Novara, Italy

**Keywords:** Gas6, TAM receptors, lung, pulmonary fibrosis, COVID-19, IPF

## Abstract

The tyrosine kinase receptors of the TAM family—Tyro3, Axl and Mer—and their main ligand Gas6 (growth arrest-specific 6) have been implicated in several human diseases, having a particularly important role in the regulation of innate immunity and inflammatory response. The Gas6/TAM system is involved in the recognition of apoptotic debris by immune cells and this mechanism has been exploited by viruses for cell entry and infection. Coronavirus disease 2019 (COVID-19) is a multi-systemic disease, but the lungs are particularly affected during the acute phase and some patients may suffer persistent lung damage. Among the manifestations of the disease, fibrotic abnormalities have been observed among the survivors of COVID-19. The mechanisms of COVID-related fibrosis remain elusive, even though some parallels may be drawn with other fibrotic diseases, such as idiopathic pulmonary fibrosis. Due to the still limited number of scientific studies addressing this question, in this review we aimed to integrate the current knowledge of the Gas6/TAM axis with the pathophysiological mechanisms underlying COVID-19, with emphasis on the development of a fibrotic phenotype.

## 1. Introduction

**Figure 1:**
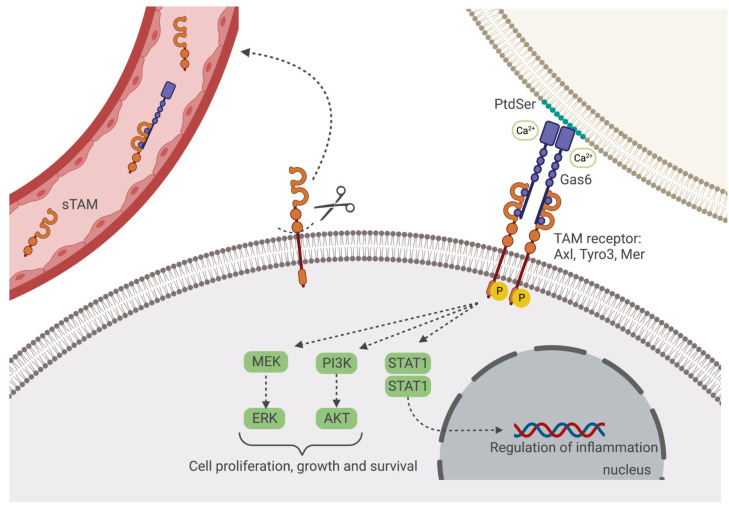
Schematic representation of Gas6/TAM binding and pathways. Gas6 binds to all the three receptors (Axl, Tyro3 and Mer) in the absence or in the presence of phosphatidylserine (PtdSer). In the latter, Gas6 interacts, through a Ca^2+^-dependent binding, with the PtdSer displayed on the extracellular surface of a plasma membrane and with the TAM-expressing cell. Upon formation of the Gas6/TAM tetrameric complex, the TAM receptor autophosphorylates its tyrosine residues in the kinase domain which activates the downstream signaling pathways, including PI3K/Akt, MEK/ERK and STAT1 pathways. The extracellular domains of TAM receptors can undergo proteolytic cleavage and be released into the bloodstream, acting as decoy receptors for Gas6. Created with BioRender.com (accessed date: 20 June 2023).

Axl, Tyro3 and Mer (gene name *Mertk*) are the tyrosine kinase receptors, which are members of the TAM family [1]. Structurally, they are composed of several types of conserved domains, including two extracellular fibronectin type III, two immunoglobulin (Ig)-like domains and one conserved kinase domain, containing a signature motif (KWIAIES) specific for TAM receptors [2]. Growth arrest-specific gene 6 (Gas6), a vitamin k-dependent protein, is the main ligand of these receptors and the only one known to activate Axl (Figure 1) [3]. Other described ligands of TAM receptors include protein S [4], tubby and tubby-like protein [5] and galectin-3 [6]. Stoichiometrically, two Gas6 and two receptors form a tetrameric complex (2:2) and both TAM receptors and their ligands are broadly expressed by multiple cell types and organs [7,8]. Gas6 is composed of a γ-carboxyglutamate (Gla)-rich domain, four epidermal growth factor-like domains and one sex hormone-binding globulin (SHBG)-like domain that contains two laminin G-like domains [9,10]. While the binding of Gas6 to the receptors is mediated by the SHBG-like domain, the Gla-rich domain has been described as particularly important for the activation of TAM receptors and is essential in the recognition of phosphatidylserine (PtdSer), a phospholipid present in the plasma membrane that is usually externalized during apoptosis or cell stress (Figure 1) [11]. 

Indeed, when an apoptotic cell externalizes PtdSer, this is recognized by a TAM-expressing phagocyte, through the mediation of Gas6 or another ligand. The activation and the consequent autophosphorylation of TAM receptors are followed by the downstream activation of several signaling cascades, such as the phosphoinositide 3 kinase (PI3K)/AKT, mitogen-activated protein kinase (MAPK) or STAT1 activation by the hybrid TAM-IFNAR [12,13]. Thus, TAM receptors are involved in multiple cell pathways and functions including immune response, inflammation and cancer progression [8,14,15,16,17]. The extracellular domains of TAM receptors can also be cleaved by metalloproteases, which inactivates the receptors, yielding soluble molecules (sAxl, sMer, sTyro3) [18,19,20,21]. sAxl is able to form a complex with Gas6 to modulate Gas6-mediated signaling by regulating the amount of ligand available for the interaction with the transmembrane receptor [22]. In serum, Gas6 concentration is approximately 0.2 nM [23]. The exact roles of soluble TAM receptors are still not fully understood but increased levels of circulating receptors and/or Gas6 have been detected in different human diseases [18,20,24,25,26,27]. 

## 2. Gas6/TAM in Lung Fibrosis

Pulmonary fibrosis is a subgroup of interstitial lung diseases (ILD), that includes a variety of parenchymal lung disorders [28]. Idiopathic pulmonary fibrosis (IPF) is the most common and severe type of ILD (reported as 17–37% of all ILD diagnosis) [29] and it is characterized by progressive fibrotic remodeling of the pulmonary parenchyma, loss of structural integrity, inflammation, impaired gas exchange and respiratory failure [30]. IPF is characterized by a histopathological pattern of usual interstitial pneumonia, that includes the presence of fibroblastic foci, small areas of active fibroblastic proliferation and excessive collagen deposition [31]. Other common types of chronic fibrosing ILD include, among others, autoimmune ILDs, chronic sarcoidosis, chronic hypersensitivity pneumonitis and diseases associated with drug exposure [28,32]. Despite the data discrepancies, the annual estimated incidence of IPF in the USA and Europe is 0.22–17.4/100,000 population [29] and a has a median survival of around 3–5 years from the time of diagnosis [29,33].

IPF is a heterogeneous rare disease whose etiology is still unclear. However, polymorphisms and gene mutations [34,35,36,37], epigenetics [38], age and sex [39,40] and environmental factors, such as exposure to cigarette smoke or metal and textile dust and farming/livestock [41], might be risk factors for its development. It is believed that IPF could be initiated by repetitive epithelial injury, ultimately leading to inflammation and fibrosis [30]. Mutations in genes involved in the normal epithelial functioning, such as mucus and surfactant-related genes, are implicated in familiar or sporadic forms of pulmonary fibrosis [35,37,42]. Similarly, a dysfunctional production of pro-inflammatory cytokines by the alveolar epithelium, such as the pro-fibrotic cytokine transforming growth factor-β (TGF-β) stimulates fibrogenesis [43]. Other growth factors such as connective tissue growth factor (CTGF), platelet-derived growth factor (PDGF) and fibroblast growth factors (FGFs) have been found upregulated in pre-clinical models of lung fibrosis and in lung tissue of patients with pulmonary fibrosis [44,45,46,47,48]. Fibroblasts are mesenchymal cells with an important role in structural support and tissue repair and, in the lung interstitium, resident fibroblasts are the most commonly identified cell type, mainly responsible for the production of extracellular matrix (ECM) [49]. During a normal process of injury–repair, fibroblasts transiently exhibit an activated myofibroblast phenotype required for the secretion of new ECM, facilitating the proliferation of alveolar epithelial type 2 cells (AEC2), an important heterogeneous population of cells acting as progenitors capable of differentiating into alveolar epithelial type 1 cells (AEC1) [50,51]. Conversely, pathological fibrosis is characterized by dysregulated proliferation and differentiation of fibroblasts into myofibroblasts that continue to deposit altered ECM, leading to aberrant epithelial repair and re-epithelialization [52]. Pirfenidone and nintedanib, the two antifibrotic drugs licensed for the treatment of IPF, mostly modulate properties of fibroblasts and myofibroblasts [53,54].

Due to their proximity to myofibroblasts and the capacity of secreting fibroblast-activating factors, alveolar macrophages might play a role in IPF pathogenesis [55]. Macrophages are immune cells that reside in all major tissues. Under acute inflammatory stimuli, circulating monocytes can migrate from the bloodstream and differentiate into macrophages. Tissue-resident macrophages exhibit elevated plasticity and during inflammation and wound healing processes, they can switch between an M1 subtype (pro-inflammatory) and a M2 (anti-inflammatory/pro-fibrotic) phenotype, when differently stimulated [56]. Even though macrophages produce matrix metalloproteinases (MMPs) that degrade ECM [57], they are also a source of TGF-β, FGF, PDGF and vascular endothelial growth factor (VEGF), that promote fibroblasts proliferation and differentiation and collagen synthesis [58,59]. Accordingly, in an animal model of bleomycin-induced lung fibrosis, the ablation of macrophages reduced pulmonary fibrosis, even though they seemed to not be involved in the early inflammatory phase of the disease development [60]. While some authors reported a shift in macrophages towards the M2 phenotype during lung fibrosis [61,62,63], others observed a general upregulation of M1 and M2 genes without a clear preference towards any of the specific macrophage subtype [64]. Human alveolar macrophages were found to overexpress CC chemokine ligand 18 (CCL18) in patients with pulmonary fibrosis and CCL18 production was negatively correlated with pulmonary function tests. Additionally, CCL18 and collagen are involved in a positive feedback loop: CCL18 enhances collagen synthesis, while collagen itself stimulates CCL18 production by macrophages [65]. Other studies also reported that monocyte-derived alveolar macrophages have a key role in the disease pathogenesis [64,66].

Although many studies have focused on the roles of the Gas6/TAM system in lung pathophysiology, mostly in tumor development and resistance to cancer therapies [16,67,68,69,70,71], the number of those assessing the contribution of the Gas6/TAM axis to the development of pulmonary fibrosis is still very limited. Mer was found to be upregulated in a sub-population of IPF macrophages, possibly being involved in the activation of IPF myofibroblasts and lung fibrosis [72]; on the other hand, Axl was associated with loss of alveolar epithelium integrity and it was identified as a negative regulator of an alveolar epithelial phenotype [73]. In another study, Axl is overexpressed in response to tobacco smoke, and it was suggested as a potential marker for smoke-associated pulmonary fibrosis [74]. Espindola and co-workers reported increased activation of the Gas6/Axl/Tyro3 pathway, both in lung biopsies and in cultured fibroblasts and mesenchymal progenitor cells from the same IPF lung tissues [75]. Gas6 and Axl transcript levels were significantly increased in IPF lungs compared to controls, and phospho-Axl, Tyro3 and α-smooth muscle action (αSMA) were detected in the fibroblastic foci of histologic sections of IPF patients. Interestingly, inhibition of TAM receptors reduced IPF fibroblast invasion and myofibroblast differentiation in vitro, and attenuated pulmonary fibrosis in humanized SCID/Bg mice injected with IPF fibroblasts. The authors also observed less hydroxyproline content in Gas6^−/−^ mice compared with wild-type mice, after continuous exposure to bleomycin [75]. Another study also reported increased levels of Axl transcripts, in a YAP-dependent manner, throughout the IPF lung tissue, particularly in epithelial cells [76]. Of note, protein S is a close structural analog of Gas6 that also binds TAM receptors [4], although with different specificities [3,77]. However, functionally, protein S is a negative regulator of the clotting cascade, while Gas6 has no major role in the coagulation process [78]. Protein S can be found in plasma in a free form or complexed to C4BP (C4b-binding protein) [79,80]. In a study on 33 IPF patients and 44 controls, no significant differences were observed in the circulating levels of free and total protein S in IPF patients compared with healthy individuals [81], while in another study, lower plasma concentrations of protein S were detected in a small cohort of 11 patients affected by IPF if compared to 20 healthy controls [82]. More recently, it was reported that 3,5,3′-triiodothyronine (T3) administration in a mice model of pulmonary fibrosis improved alveolar regeneration, in a process involving protein S-TAM signaling [83].

## 3. Gas6/TAM System in Viral Infection

**Figure 2:**
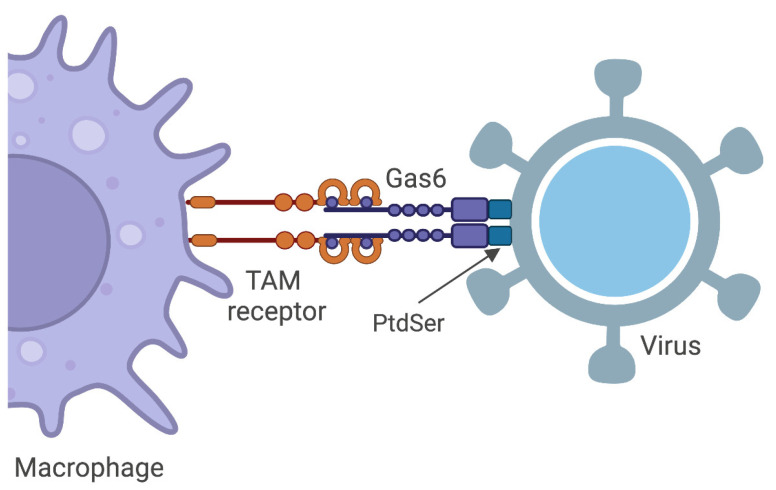
Upon the binding of Gas6, both to phosphatidylserines (PtdSer) exposed by enveloped virus and to TAM receptors, viral entry is facilitated. The PtdSer resembles the one expressed on the apoptotic cell surface that usually binds to Gas6 and TAM receptors on the surface of dendritic cells, macrophages and other phagocytes during the phagocytosis. In the same way, Gas6 and TAM receptors recognize PtdSer and act as entry receptors through a process called apoptotic mimicry. Created with BioRender.com (accessed date: 20 June 2023).

It has been reported that enveloped viruses such as Ebola and Vaccinia viruses are able to manipulate host cells mediating cell entry and promoting infection through TAM receptors and apoptotic mimicry [84]. In particular, the PtdSer, a marker for apoptosis on the membranes, disguises viruses as apoptotic bodies, leading to the engulfment of infectious particles through cell clearance mechanisms [85]. It has been demonstrated that Gas6 is able to bind PtdSer on the virion surface and, through the interaction with TAM receptors, bridges the virus to the cell surface of macrophages and other phagocytes, inducing viral internalization (Figure 2) [86,87]. After the binding between virus, Gas6 and TAM receptor, the latter promotes clathrin-mediated endocytosis or the macropinocytosis of viruses [88].

The versatile role of the Gas6/TAM system in viral infection is supported by its involvement, not only in mediating or facilitating viral entry, but also in other major functions [88]. Indeed, Gas6 is able to inhibit the toll-like receptor-triggered inflammatory responses by binding and activating the TAM receptors [89]. Sun et al. highlighted the role of Tyro3, Axl and Mer as negative regulators of TLR3 signaling in Sertoli cells. The activation of TLR3 results from one side in the activation of TLR3-TRIF-IRF3 signaling and type I interferons induction, and from another side in the activation of TLR3-TRIF-NF-κB signaling and pro-inflammatory cytokines production. The binding between Gas6 and TAM receptors allows the activation of STAT1/2, their translocation into the nucleus and the promotion of the transcription of the suppressor of cytokine signaling (SOCS)1 and SOCS3 which in turns inhibit TLR3-TRIF-IRF3 and TLR3-TRIF-NF-κB signaling blocking the production of type I IFNs and pro-inflammatory cytokines [90].

Merteens et al. described that Axl and its ligand Gas6 play a major role in ZIKA virus (ZIKV) infection in human glial cells. The binding between ZIKV-Gas6-Axl results in the downregulation of different interferons including IFN-β, IFN-λ1, IFN-λ2 and pro-inflammatory cytokines such as tumor necrosis factor α (TNF-α), interleukin 6 (IL-6) and interleukin 1β (IL-1β) [91]. Moreover, it was also observed that non-enveloped viruses might activate TAM receptors, in particular, the binding between the Axl receptor, Gla domain of Gas6 and Ad fiber protein contributes to adenovirus vaccine vector (AdV) immunogenicity by reducing the IFN response stimulated by HAdV-5C vectors and enhancing HAdV-5C vector-encoded transgene expression [92]. Additionally, Miner et al. demonstrated the presence of a substantial vulnerability to infection with neuroinvasion in Mer-lacking mice when infected with West Nile and La Crosse viruses. The infection in these mice resulted in increased blood–brain barrier permeability enhancing viral spreading in the brain. Indeed, the activation of Mer together with IFN-β is capable of preserving the integrity of the blood–brain barrier through the stabilization of thigh junction proteins (Claudin5, Occludin, ZO1), preventing viral transition across brain microvascular endothelial cells and as a consequence, restricting neuroinvasion [88,93]. Finally, Persaud et al. found that the Axl receptor plays a crucial role in the ZIKV infection of human fibroblasts, serving as an entry point, and that the productive infection requires endocytosis and delivery of the virus to acidified intracellular compartments [94].

## 4. Involvement of Gas6/TAM Axis in COVID-19

Coronaviruses (CoVs) are a group of RNA viruses taxonomically belonging to the subfamily *Coronavirinae*, family *Coronaviridae* and order *Nidovirales* which are genetically classified into four major genera: *Alphacoronavirus*, *Betacoronavirus*, *Gammacoronavirus* and *Deltacoronavirus* [95,96,97]. Among them, *Alphacoronavirus* and *Betacoronavirus* are commonly found in humans and other mammals, whereas the former two genera largely infect avian species [98]. Over the last two decades, two zoonotic outbreaks of *Betacoronaviruses* have occurred as the result of spillover events, severe acute respiratory syndrome coronavirus (SARS-CoV) in 2002–2003 [99] and Middle East respiratory syndrome coronavirus (MERS-CoV) in 2012 [100]. During December 2019, the first cases of pneumonia due to the infection of severe acute respiratory syndrome coronavirus 2 (SARS-CoV-2) were reported in Wuhan, the capital city of Hubei province in China [101]. SARS-CoV-2 is the etiological agent of the acute respiratory disease, named as coronavirus disease 2019 (COVID-19), which has evolved into a global concern due to its fast diffusion [102]. Indeed, within a few months, the virus spread across the world causing a pandemic officially declared by World Health Organization (WHO) in March 2020 [103]. The SARS-CoV-2 genome shares a 79% and 50% sequence identity with SARS-CoV and MERS-CoV genome, respectively [104]. Despite the fact that SARS-CoV-2 seems to be less lethal compared to SARS-CoV or MERS-CoV, its transmissibility is higher and the predominant modes of transmission between humans have been recognized to be through droplets of respiratory mucus secretion, close unprotected contact between individuals during activities such as speaking, breathing, coughing, sneezing or indirect contact [105]. SARS-CoV-2 is an enveloped, single-stranded, positive-sense RNA of ~30-kb with 5′-cap structure and 3′-poly-A tail [106]. It has a crown-like appearance and a diameter approximately ranging from 60 to 140 nm [107]. Two-thirds of the SARS-CoV-2 genome is occupied by ORF1a and ORF1b which encode for two polyproteins, pp1a e pp1ab, that are processed by viral proteases, belonging to papain-like protease, in order to obtain 16 non-structural proteins (nsps) [108]. Furthermore, the remaining one-third of the genome contains overlapping ORFs encoding for accessory proteins (3a, 3b,6, 7a, 7b, 8a, 8b, 9b) and four major structural proteins: spike protein (S), membrane protein (M), nucleocapsid protein (N) and envelope protein (E) [109]. Coronavirus spike glycoproteins are homotrimeric surface glycoproteins that can be divided into two functional subunits (S1 and S2). The surface-exposed S1 includes the receptor-binding domain (RBD) that specifically recognizes the angiotensin-converting enzyme 2 (ACE2) receptor in the host cell, which mediates the viral entry [110]. In turn, S2 domain is involved in membrane fusion [111]. The ACE2 receptor is predominantly expressed by lung alveolar epithelial cells, bronchial transient epithelial secretory cells, pneumocytes, myocardial cells, intestinal enterocytes, vascular endothelial cells and smooth muscle cells in humans [112]. More recently, it was proposed that some cells might possess ACE2-independent alternative receptors that are still able to mediate SARS-CoV-2 entry [113]. SARS-CoV-2 infection may lead to a variety of clinical manifestations ranging from asymptomatic or mild to moderate and severe cases [114]. The clinical symptoms of COVID-19 infection arise after 5–6 days of incubation; however, this period also depends on age and the individual immune system [115]. In addition, females are less susceptible to severe infection compared to males who, instead, are more vulnerable [116]. Even though in the vast majority of patients SARS-CoV-2 infection results into flu-like symptoms including fever, shortness of breath, rhinitis, dry cough, fatigue, dyspnea and, additionally, loss of smell and taste [117], in some patients, SARS-CoV-2 leads to severe pneumonia, acute respiratory distress syndrome (ARDS) and lung injuries [110]. Nonetheless, given the fact that ACE2 is expressed in different extrapulmonary tissues, the infection may also result in extrapulmonary manifestations. These conditions include neurologic, renal, hepatic, gastrointestinal, cardiac, endocrine and dermatological manifestations [118]. It has been described that, on average, about 80% of COVID-19 patients remain asymptomatic or experience mild or moderate symptoms, 15% of them develop severe pneumonia and 5% progress into acute respiratory distress syndrome or multiple organ failure. However, this picture has changed over time thanks to the development of vaccines and the arrival of new variants [119]. Furthermore, patients with pre-existing conditions, such as hypertension, diabetes and cardiovascular diseases, are more prone to rapidly develop ARDS, heart failure, kidney damage, septic shock, metabolic acidosis, coagulation and liver dysfunction and secondary infection, that eventually may result in death [120]. On the contrary, the available vaccines have played a crucial role in positively modulating the severity of the disease and the worst outcomes [121].

### 4.1. Innate Immune System

During antiviral innate immune response, macrophages, monocytes, natural killer cells, dendritic cells and neutrophils recognize, through pattern recognition receptors (PRRs), peculiar molecular viral structures called pathogen-associated molecular patterns (PAMPs) and damage-associated molecular patterns (DAMPs) produced by virus-infected cells leading to the initiation of the protective responses [122,123]. This recognition is responsible for the starting of the inflammatory response through the recruitment of adaptor proteins capable of activating the downstream signaling pathways and transcription factors that induce the expression of genes involved in the release of several pro-inflammatory cytokines and chemokines. Pro-inflammatory cytokines are able to activate and recall immune cells which migrate to the site of infection promoting further inflammation and generating a pro-inflammatory feedback loop [124,125]. The pro-inflammatory cytokine release is considered as a beneficial mechanism to destroy the invading virus promoting local coagulation and limiting tissue damage [126]. Nonetheless, when the cytokine production is excessive, it has also detrimental effects on the organism that can be more dangerous compared to the original stimulus [127].

**Figure 3:**
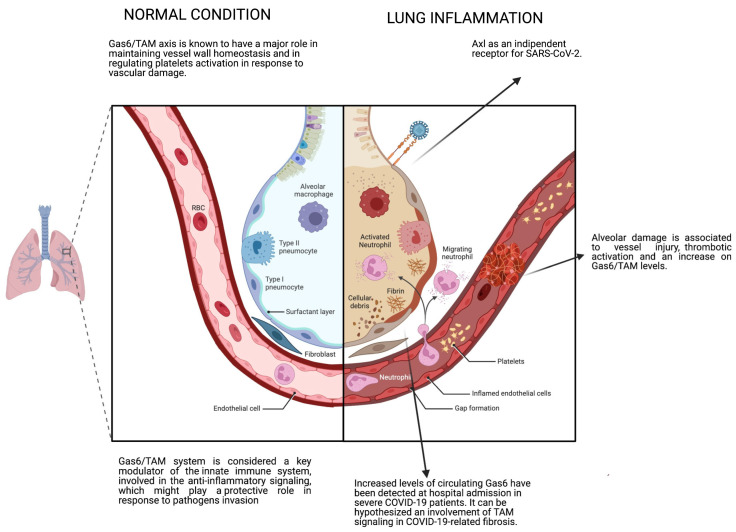
Gas6 and TAM receptor roles in homeostasis and COVID-19 pathogenesis. Under normal conditions, the Gas6/TAM system regulates several biological mechanisms, including anti-inflammatory response, modulation of the immune system and maintenance of vessel wall homeostasis. However, during COVID-19 infection and lung inflammation, Axl has been reported to enhance infection by promoting viral entry, and circulating levels of Gas6 and sTAM have been associated with disease severity. Created with BioRender.com (accessed date: 20 June 2023).

The TAM family might be involved in COVID-19 pathogenesis at different levels. TAM might not only contribute to the viral internalization into epithelial cells of the airways but may also be required for adaptive immunity and damage resolution [128,129]. In fact, Axl itself was proposed as a candidate receptor for SARS-CoV-2 [130] (Figure 3) and therapeutical approaches targeting Axl have been tested as potential treatments for COVID-19 [131,132]. In one recent study, the authors reported that secondary RNA structures, called RNA G-quadruplex (RG4), can be found within SARS-CoV-2 host factors (*Axl*, *Ace2*, *Furin* and *Tmprss2*) [133]. These structures can regulate gene expression and translation, and pharmacological RG4 stabilization with topotecan- and berbamine-reduced Axl protein levels and be able to prevent SARS-CoV-2 pseudovirus entry in vitro and in vivo. The Gas6/TAM system is also considered a key modulator of the innate immune system, involved in the anti-inflammatory signaling, which might play a protective role in response to pathogen invasion [134,135]. Indeed, components of this axis are increased in a wide spectrum of inflammatory conditions [18,19,136,137,138]. sAxl, sMer and sTyro3, in this context, can contribute to the modulation of inflammatory responses acting as scavenger receptors for TAM ligands [138]. In COVID-19, plasma Gas6 and sTAM levels have been shown to reflect disease severity and have been identified as possible early biomarkers of disease prognosis. Morales and colleagues demonstrated that SARS-CoV-2-positive patients, enrolled during the first pandemic wave, exhibited higher Gas6 serum levels that gradually increased together with disease severity. They also reported that deceased SARS-CoV-2-positive patients showed higher plasma levels of sAxl and sMer at the time of hospital admission, suggesting that higher concentrations of these tyrosine kinases at emergency ward entry could be predictors of the worse prognosis [26]. These data are in line with another study from Tonello et al., according to which higher baseline plasma Gas6 concentration in mild to moderate COVID-19 patients predicted a more severe disease evolution. In addition, sMer levels, measured at the baseline and after 7 days of hospital stay, were lower in patients with a more favorable disease evolution, even though the latter results were not supported after correction for demographic and severity variables [27]. Galectin-3, another ligand associated to Mer and Tyro3 activation, was significantly increased in COVID-19 patients who developed pneumonia, and positively correlated with several inflammatory and tissue injury markers [139,140].

### 4.2. Coagulation and Vascular Functions

TAM signaling has also been referred to in coagulopathies associated to COVID-19 [141]. Gas6/TAM axis is known to have a major role in maintaining vessel wall homeostasis and in regulating platelet activation in response to vascular damage and in order to repair the endothelium [142,143,144]. Following vessel injury, activated platelets expose PtdSer on the cell surface, recruiting different actors; among them, Gas6 is secreted by endothelial cells and upregulates the expression of adhesion molecules, including P-selectin, vascular cell adhesion protein 1 (VCAM-1) and intercellular adhesion molecule 1 (ICAM-1), ultimately leading to the recruitment of platelets and leukocytes at the endothelium [145,146]. TAM signaling is known to be involved in platelet aggregation and thrombus stabilization; in particular, TAM is able to induce the phosphorylation of β3 integrin through PI3K pathway promoting aggregation [147]. In critically ill COVID-19 patients, alveolar damage is associated with vessel injury and thrombotic activation and an increase in Gas6/TAM levels in these patients might explain the abnormal coagulation parameters linked to COVID-19 [148] (Figure 3). On the contrary, inhibition of the Gas6/TAM system was shown to decrease platelet activation responses and was able to prevent arterial and venous thrombosis in in vivo studies [23,149,150], thus representing a possible therapeutic target for novel anti-platelet agents [146]. Lemke et al. hypothesized that the blood-clotting formation and the exaggerated immune reaction could be linked to anticoagulant protein S. According to their hypothesis, the dysregulated clot formation may lead to protein S consumption. Lower levels of protein S might downregulate Mer signaling, leading to a dysregulated release of proinflammatory cytokines [151].

### 4.3. Fibrosis Development

As previously described, pulmonary fibrosis can be initiated as a result of multiple events or pathologies. In particular, viral infections have been associated with the triggering or aggravation of fibrotic conditions [152,153]. As reviewed by Huang and Tang, pulmonary fibrosis following viral infection might be initiated (1) as a direct consequence of the lung damage and abnormal wound healing caused by the virus and/or (2) by immune-mediated injury and the activation of pro-inflammatory and pro-fibrotic signaling [154]. Over recent years, several reviews have been published addressing the effects of COVID-19 on lung function [155,156,157,158,159,160]. The severe cases of COVID-19 might result in ARDS, and a subset of ARDS survivors will develop lung fibrosis [155,161]; on the other hand, the pre-existence of ILD is by itself a disease risk that increases the odds of severe disease and death from COVID-19 [162]. Lung fibrotic-like changes have been observed in more than one third of 114 patients recovered from severe COVID-19, within 6 months of disease onset [163]. In another study from Zhou and coworkers, fibrotic streaks were observed in 56.5% of patients with COVID-19 pneumonia [164]. Even though the etiology of several types of pulmonary fibrosis might differ, many disease-related mechanisms are commonly shared between chronic diseases, such as IPF, and ARDS-related fibrosis. The damage of the pulmonary epithelium is usually the trigger for the subsequent cascade of events. It has been hypothesized that SARS-CoV-2 might have a preference for infecting AEC2, due to their high expression of ACE2, an essential receptor for the entry of the virus [165,166]. This viral-mediated cell death would be followed by abnormal re-epithelialization, endothelial injury, infiltration of fibroblasts and inflammatory cells, and an overall hyperactive immune response and excessive production of cytokines, leading to pulmonary fibrosis [167]. Average serum levels of cytokines such as IL-2, IL-7, IL-8, IL-10, IL-17, IFN-γ and TGF-β were significantly higher in COVID-19 patients compared to healthy subjects [168,169], and transcriptional analysis showed that SARS-CoV-2 spike modulates the expression of genes involved in the regulation of ECM and TGF-β signaling pathways [170]. Importantly, differences were observed in pro-fibrotic gene expression and protein profiles of non-resolvable COVID-19 lung tissues compared to IPF tissues, suggesting that some molecular features are specific for each pathology [171]. Since the Gas6/TAM axis has been already related with the development of liver and pulmonary fibrosis in other pathologies [75,172,173], and increased levels of circulating Gas6 have been detected at hospital admission in severe COVID-19 patients [27], an involvement of TAM signaling in COVID-19-related fibrosis can be hypothesized. The use of mechanical ventilation during hospitalization also carries its own risks, such as the occurrence of ventilator-associated lung injury, and it has been connected to the development or exacerbation of post-ARDS fibrosis [155]. To date, it is not possible to reliably estimate the long-term incidence of fibrosis progression after COVID-19 recovery, but it was assessed for another coronavirus with similar clinical course and pathogenic features as the one induced by SARS-CoV-2. Indeed, a 15-year observational study based on lung pathology after SARS revealed that while most of the SARS patients with fibrotic lung damage have recovered within the first year after the infection, in about 20% of those patients, fibrosis progressed in 5–10 years [174].

### 4.4. Long COVID

Nowadays, it is well consolidated that some COVID-19 survivors may experience physical and neuropsychiatric symptoms which persist for several months after the initial recovery [175,176,177,178]. The term long COVID refers to these sequelae and long-term complications that have been described following COVID-19 and cannot be explained by an alternative diagnosis [179,180]. In contrast with the acute phase of the disease in which females had a reduced risk of developing severe disease, in long COVID they are disproportionally affected compared to males [181]. Although the underlying pathogenetic mechanisms involved in long COVID are not clearly understood [182], it has been suggested that these long-lasting conditions may be associated with a pro-inflammatory status boosted by cytokines, pro-coagulative conditions, direct tissue damage due to substantial alteration of the vessel barrier integrity and endothelial injury, immune system dysregulation, hypercoagulability and the persistence of a viral reservoir [183,184,185]. As far as we know, the involvement of the Gas6/TAM system in the pathogenesis of long COVID has not yet been evaluated, but it would be interesting to assess the possible role of this pleiotropic axis in the development of long-term sequelae. More recently, we first showed that the levels of sAxl and Gas6 in post-COVID-19 subjects, one year after hospital discharge, were still associated with the class of severity reported during the acute phase of the disease. Based on our results, the Gas6/TAM system has not been associated with persistent symptoms among this population. Interestingly, lower levels of Gas6 and sAxl were also associated with patients who had a history of hair loss following COVID-19 [186].

## 5. Conclusions

The Gas6/TAM system has gained attention in the last few years given its involvement in several human pathologies [18,74,75,172,187,188,189]. Some authors have recently highlighted the role of the Gas6/TAM system as potentially relevant also in COVID-19 pathogenesis. The ACE2 receptor has an important role in SARS-CoV-2 infection, but alternative receptors, including Axl, have also been studied as entrance routes for the virus. This fact, together with the crucial function of TAM-related signaling in the regulation of inflammation and the observed predictive role of circulating Gas6 and TAM with disease severity, has confirmed the importance of further investigating this system in the COVID-19 context. Additionally, substantial evidence suggests that Gas6 and TAM have an important role in the interface between inflammation and fibrosis and are involved in the development of multiple fibrotic diseases, namely in liver and lung. Regarding the latter, even though the literature available is still scarce, there are some interesting reports associating Gas6/TAM with the pathogenicity of fibrotic lung diseases, in particular IPF. In this sense, we highlight the importance of further exploring this system in other fibrotic conditions, such as in connective tissue disease-associated ILD, and in COVID-related fibrosis. The development of reliable fibrotic lung disease models might, therefore, allow the identification of potentially overlaying mechanisms responsible for disease pathogenesis. Furthermore, exploring the Gas6/TAM system as a player in disease development and progression could provide new therapeutic strategies for COVID-19 patients or patients experiencing other fibrotic conditions. In particular, the administration of TAM inhibitors may be examined not only in terms of SARS-CoV-2 entry and replication but also on how it affects inflammation and long-term fibrotic lung complications.

## Data Availability

Not applicable.
